# Malaria investigation and treatment of children admitted to county hospitals in western Kenya

**DOI:** 10.1186/s12936-016-1553-6

**Published:** 2016-10-18

**Authors:** Beatrice I. Amboko, Philip Ayieko, Morris Ogero, Thomas Julius, Grace Irimu, Mike English, on behalf of Clinical Information Network authors

**Affiliations:** 1 Kenya Medical Research Institute/Wellcome Trust Research Programme, P.O. Box 43640, Nairobi, 00100 Kenya; 2 Department of Paediatrics and Child Health, University of Nairobi, Nairobi, Kenya; 3 Nuffield Department of Medicine, Oxford University, Oxford, UK

**Keywords:** Malaria, Under-fives, Children, Anti-malarials, Malaria case management, Inpatient

## Abstract

**Background:**

Up to 90 % of the global burden of malaria morbidity and mortality occurs in sub-Saharan Africa and children under-five bear a disproportionately high malaria burden. Effective inpatient case management can reduce severe malaria mortality and morbidity, but there are few reports of how successfully international and national recommendations are adopted in management of inpatient childhood malaria.

**Methods:**

A descriptive cross-sectional study of inpatient malaria case management practices was conducted using data collected over 24 months in five hospitals from high malaria risk areas participating in the Clinical Information Network (CIN) in Kenya. This study describes documented clinical features, laboratory investigations and treatment of malaria in children (2–59 months) and adherence to national guidelines.

**Results:**

A total of 13,014 children had a malaria diagnosis on admission to the five hospitals between March, 2014 and February, 2016. Their median age was 24 months (IQR 12–36 months). The proportion with a diagnostic test for malaria requested was 11,981 (92.1 %). Of 10,388 patients with malaria test results documented, 8050 (77.5 %) were positive and anti-malarials were prescribed in 6745 (83.8 %). Malaria treatment was prescribed in 1613/2338 (69.0 %) children with a negative malaria result out of which only 52 (3.2 %) had a repeat malaria test done as recommended in national guidelines. Documentation of clinical features was good across all hospitals, but quinine remained the most prescribed malaria drug (47.2 % of positive cases) although a transition to artesunate (46.1 %) was observed. Although documented clinical features suggested approximately half of positive malaria patients were not severe cases artemether-lumefantrine was prescribed on admission in only 3.7 % cases.

**Conclusions:**

Despite improvements in inpatient malaria care, high rates of presumptive treatment for test negative children and likely over-use of injectable anti-malarial drugs were observed. Three years after national policy change, there is a gradual transition to artesunate. Continued efforts to support improved routine inpatient malaria care through dissemination and implementation of guidelines, and access to recommended drugs are needed together with improved capacity of hospitals to investigate other causes of severe illness in children. Efforts to improve clinical information could help track progress.

## Background

Worldwide estimates indicate that three-quarters of malaria infections involving children under-5 years occur in sub-Saharan Africa [1]. Malaria case management backed up with rapid diagnostics or laboratory investigations is the main strategy for treating malaria in low-income countries [2]. The case management strategy promoted by the World Health Organization (WHO) is based on well-described clinical features of severe malaria [3–6] and effectiveness trials of recommended treatments [7]. The WHO in 2010, revised its treatment policy to recommend universal testing for all suspected malaria cases prior to treatment, and replacement of the then existing first-line anti-malarial drugs with new therapies in both outpatient and inpatient malaria treatment [8, 9]. These recommendations were adopted in Kenya in 2010 [10] and in 2012 [11], but although reports of outpatient malaria treatment change from sulfadoxine-pyrimethamine (SP) to artemisinin-based combination therapy (ACT) show major improvements in quality of malaria case management, significant challenges including drug stock-outs still persist [12, 13].

In the inpatient setting such assessments are rare. Existing primary studies on inpatient malaria treatment suggest a need to consider the wider clinical decision-making context in addition to simple dissemination of clinical guidelines [14, 15]. Similarly, systematic reviews indicate that clinical guidelines have a variable effect on uptake of new drugs and diagnostics in secondary care (equivalent to County Hospitals in Kenya). Among the factors that have been postulated to have an effect on guideline implementation especially with regard to uptake of new drugs and diagnostics is strong professional networks [16]. Descriptive analysis of inpatient childhood malaria treatment practices in Kenyan hospitals in the period following treatment guidelines amendments in Kenya was done. Data obtained from the recently established paediatric Clinical Information Network in Kenya were used to examine adoption of malaria guidelines which recommend parasitological diagnosis of malaria, adherence to test results, and use of injectable artesunate and not quinine in treatment [9, 17, 18].

## Methods

### Study design and setting

This was a descriptive cross sectional study. A subset of the data from five of the 14 hospitals participating in the CIN collected during a 24-month period (March, 2014 to February, 2016) was used. All the five hospitals are located in the western Kenya, a high malaria prevalence region and serve predominantly poor populations.

### Participants

All children aged between 2 and 59 months admitted to the paediatric wards in the participating hospitals with a diagnosis of malaria were eligible for inclusion in the study.

### Data collection and management

Since its establishment in October 2013 CIN has supported data capture from paediatric medical records that include a standardised admission record form using the non-proprietary Research Electronic Data Capture (REDCap) tool in all participating hospitals [17]. Support provided to hospitals participating in the network includes an additional post for a clerical assistant in each hospital and provision of one basic computer. Data collection is done on a daily basis by the clerk from medical records of all admitted patients in paediatric wards using detailed standard operating procedures under the joint supervision of the hospital medical records department and the CIN team. Data quality is promoted through the use of range and quality checks in the electronic data tool and error checking procedures. The details of data management have been previously published [17]. In brief, a process for validating the data collected at each site is conducted bimonthly through conducting double entry for selected records. During the validation at least 140 records previously entered by CIN data clerks in the 2-month period prior to the validation are re-entered by an independent trained data collection team. Ten records are selected at random for re-entry at each hospital (giving a total of 140 records across the network). Analysis is then done to validate data using R scripts that conduct field by field comparison for all fields in the database. A total concordance score is then calculated for each record and feedback is immediately given to data clerks in areas where there is discordance between the two sets of entries. During the most recent round of data validation the overall concordance was 85 %.

### Statistical analysis

No formal sample size calculations were undertaken, but data on over 13,000 children aged between 2 and 59 months with an admission diagnosis of malaria were considered adequate to provide reasonably precise estimates around malaria case management practices. Descriptive analysis of the malaria admissions pooled across all hospitals and for each hospital included calculating frequencies, relative frequencies and medians (IQR) for categorical and continuous variables, respectively. Indicators of good practice for five key areas of inpatient malaria case management spanning clinical assessment, laboratory investigation and treatment are defined in Table 1 and proportions of patients managed according to the current malaria guidelines are reported. The proportion of children for whom six specific features of malaria were assessed and documented in medical records was calculated. The level of adherence to parasitological testing recommendations was determined by calculating the percentage of malaria admissions with parasitological confirmation at the time of initiating anti-malarial drugs. Finally, for parenteral anti-malarials the proportion with the correct dosages based on body weight (drug dose per kg body weight), allowing for the dosage to be within 20 % of recommended dosage (guideline dosage ± 20 %) were calculated. No attempts were made to impute data where these data were missing for calculating specific indicators (for example if the child’s weight was missing correctness of drug dosage could not be estimated). In addition, the following indicators were used to assess the routine practice of using laboratory testing to guide management; proportion with laboratory requests of haemoglobin level, blood chemistry and glucose level. To identify evidence that clinicians considered other causes of febrile illnesses the proportion of malaria patients with the following tests requested: HIV status, blood culture/lumbar culture, x-rays and urine tests and the proportions prescribed antibiotics were calculated. All analyses were conducted using R statistical software (version 3.2.2) [19].

**Table 1:** Malaria policy recommendations and the indicators used to assess implementation and adherence

Policy recommendation	Indicator
All key clinical features of severe malaria should be assessed and documented	Proportion among the children with a diagnosis of malaria with documented assessment of the following clinical features: level of consciousness (AVPU scale), deep acidotic breathing or chest in-drawing (respiratory distress), fever, ability to drink, convulsions and pallor
All suspected malaria cases without a sign of severe disease should have parasitological confirmation by diagnostic testing before initiating treatment	Proportion of patients with a diagnosis of malaria with a malaria test ordered on the admission date and with the results recorded in the medical notes or laboratory register
Malaria positive cases should be prescribed anti-malarial drug	Proportion with malaria positive test prescribed anti-malarials Proportion with malaria negative test prescribed anti-malarials Type of anti-malarial drugs prescribed
Anti-malarial drug dosages; Quinine loading dose of 20 mg, maintenance dose of 10 mg and artesunate at 2.4 mg per kg body weight with a 20 % margin of error	Proportion with malaria positive prescribed quinine or artesunate and proportions with correct dosing
Management of malaria negative; anti-malarial drugs should be withheld in non-severe malaria cases with a negative test; those with signs of severe malaria be started on presumptive treatment but testing should be repeated	Proportion of patients with admission diagnosis of severe malaria but a negative admission test and given anti-malarials who had repeat testing after admission

## Ethical consideration

Scientific and ethical approval for the study was obtained from the Kenya Medical Research Institute Scientific and Ethics Review Unit as part of the CIN study (SSC protocol no. 2465) that provides for use of de-identified patient data obtained through retrospective review of medical records without individual informed consent for audit and quality improvement purposes.

## Results

### Malaria diagnosis and clinical features

**Fig. 1:**
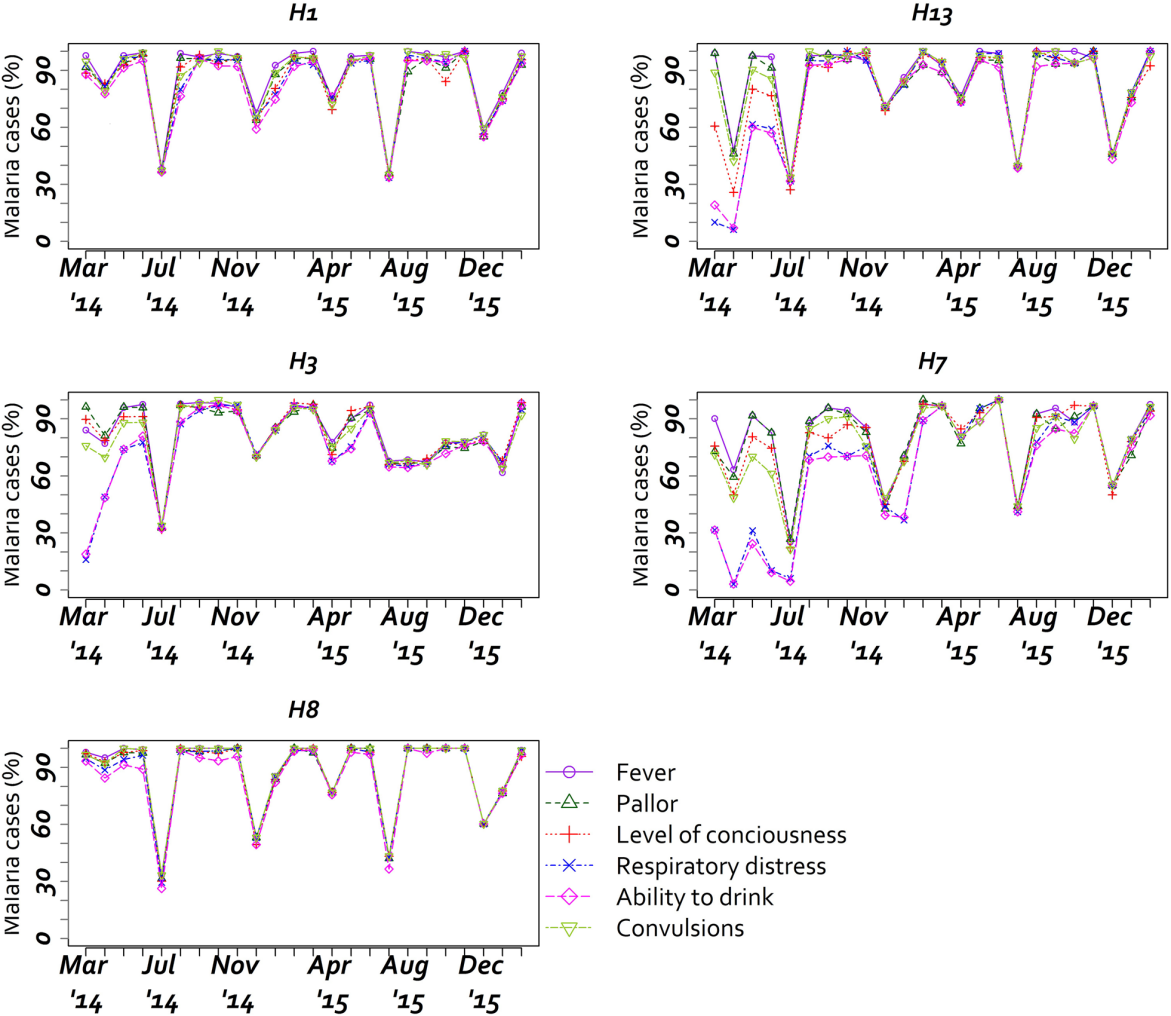
Temporal trend in documentation of clinical signs from March, 2014 to February, 2016

During the 24-month period, there were 19,419 acute medical admissions out of which 13,014 (67 %) children had admission diagnosis of malaria (range 1597–4572 per hospital). The median age of malaria admissions across all hospitals was 24 months (interquartile range 12–36), and 53 % were male. Fever was a common presenting feature, 91.6 % of malaria admissions with complete documentation had fever (from 84.8 to 95.7 % per hospital). Documentation of the specific guideline recommended signs of severe malaria (fever, pallor, altered consciousness level, respiratory distress, ability to drink and convulsions) within the medical records of malaria admissions ranged from 10 to 100 % for the specific signs across hospitals (Fig. 1), and rapid improvements in documentation of malaria features was noted in three hospitals upon joining the CIN. Documentation of the five signs was 70 % with a range of 47.2–83.6 % across the hospitals. WHO defined severe malaria (characterized by the occurrence of danger signs of severe illness in a child with malaria diagnosis) was common with 49.4 % of malaria admissions presenting with at least one danger sign. The specific danger signs for malaria were present in between 8.1 and 34.8 % of admissions seen during the entire period: convulsions (34.8 %), respiratory distress (14.8 %), severe pallor (14.6 %), and altered level of consciousness (8.1 %) (Table 2).

**Table 2:** Demographic characteristics and clinical signs of childhood malaria in children admitted to five Kenyan County Hospitals

Indicator	Overall	Hospitals				
		H1	H3	H7	H8	H13
Number of malaria cases	13014	2587	4572	1695	2563	1597
Male (%)	6880/13014 (52.9)	1388/2587 (53.7)	2430/4572 (53.1)	877/1695 (51.7)	1361/2563 (53.1)	824/1597 (51.6)
Age in months, median (IQR)	24 (12–36)	20 (12–36)	23 (12–36)	20 (11–36)	15 (9–27)	24 (12–36)
All clinical features documented	9104/13014 (70.0)	2013 (77.8)	3169 (69.3)	800 (47.2)	2143 (83.6)	979 (61.3)
Malaria admissions with fever^a^ (%)	10063/10981 (91.6)	2132/2255 (94.5)	3527/3854 (91.5)	1260/1317 (95.7)	1890/2229 (84.8)	1254/1326 (94.6)
Malaria admissions with at least one danger sign^b^ (%)	6427/13014 (49.4)	1412/2587 (54.6)	2204/4572 (48.2)	675/1695 (39.8)	1185/2563 (46.2)	951/1597 (59.5)
Altered level of consciousness (%)	863/10600 (8.1)	195/2160 (9.0)	288/3828 (7.5)	74/1227 (6.0)	145/2201 (6.6)	161/1184 (13.6)
Inability to drink (%)	1264/9514 (13.3)	369/2111 (17.5)	244/3370 (7.2)	137/857 (16.0)	272/2121 (12.8)	242/1055 (22.9)
Respiratory distress (%)	1435/9692 (14.8)	266/2161 (12.3)	405/3373 (12.0)	61/895 (6.8)	539/2185 (24.7)	164/1078 (15.2)
Severe pallor (%)	1583/10807 (14.6)	383/2180 (17.6)	795/3848 (20.7)	171/1281 (13.3)	103/2202 (4.7)	131/1296 (10.1)
Convulsions (%)	3699/10625 (34.8)	775/2205 (35.1)	1227/3721 (33.0)	427/1196 (35.7)	555/2223 (25.0)	715/1280 (55.9)

*IQR* interquartile range

^a,b^The denominator for proportion with clinical signs of malaria where the respective sign was documented

### Investigation of admissions with clinical malaria

Of all the 13,014 admissions with clinical malaria, 11,981 (92.1 %) had a malaria test ordered during admission (range per hospital 82.5 to 98.7 %). Further, among these children with a malaria test requested 10,388/11,981 (86.7 %) had malaria test results documented in the clinical notes (range per hospital 66.6–99.3 %).

The majority, 8050/10,388 (77.5 %) of documented malaria test results were positive for the malaria parasite. For those with negative malaria tests still treated presumptively, 52/1613 (3.2 %, hospitals’ range 0–17.6 %), had malaria diagnostic tests repeated (Table 3). To guide management decisions in the children with malaria diagnosis (N = 13,014) the following tests were done; haemoglobin level 4626 (35.5 %), blood chemistry 178 (1.4 %) and blood glucose 333 (2.6 %). Among the children documented to have severe pallor, 1232/1583 (77.8 %) had haemoglobin level checked. Other diagnostic tests done included: HIV status ascertained (39.6 %), microbiology (blood culture or lumbar puncture for CSF culture and other microscopic and biochemical CSF investigations) (8.5 %), urine tests (1.9 %) and x-rays (2.5 %).

**Table 3:** Malaria testing and treatment practices among children admitted to five Kenyan County Hospitals: March, 2014 to February, 2016

Indicator	Overall	H1	H3	H7	H8	H13
Total malaria admissions (N)	13014	2587	4572	1695	2563	1597
*Malaria investigations*						
Proportion with malaria test requested, n/N (%)	11981/13014 (92.0)	2533/2587 (97.9)	3770/4572 (82.5)	1596/1695 (94.2)	2505/2563 (97.7)	1577/1597 (98.7)
Malaria test results available, n/N (%)	10388/11981 (86.7)	2516/2533 (99.3)	2510/3770 (66.6)	1481/1596 (92.8)	2327/2505 (92.9)	1554/1577 (98.5)
Positive malaria test in those with results documented, n/N (%)	8050/10388 (77.5)	1902/2516 (75.6)	2046/2510 (81.5)	1162/1481 (78.5)	1609/2327 (69.1)	1331/1554 (85.6)
Positive malaria test in all children diagnosed with malaria, n/N (%)	8050/13014 (61.9)	1902/2587 (73.5)	2046/4572 (44.8)	1162/1695 (68.6)	1609/2563 (62.8)	1331/1597 (83.3)
Diagnosis of severe malaria with positive test, n/N (%)	5243/8050 (65.1)	1174/1902 (61.7)	1701/2046 (83.1)	699/1162 (60.2)	793/1609 (49.3)	876/1331 (65.8)
*Antimalarial prescription*						
Proportion with positive test prescribed anti-malarial drug, n/N (%)	6745/8050 (83.8)	1642/1902 (86.3)	1760/2046 (86.0)	909/1162 (78.2)	1347/1609 (83.7)	1087/1331 (81.7)
Proportion with negative test prescribed anti-malarial drug, n/N (%)	1613/2338 (69.0)	479/614 (78.0)	372/464 (80.2)	169/319 (53.0)	445/718 (62.0)	148/223 (66.4)
Proportion of negative test treated presumptively with repeat testing, n/N (%)	52/1613 (3.2)	7/479 (1.5)	0	1/169 (0.6)	18/445 (4.0)	26/148 (17.6)
Proportion with no malaria test treated presumptively, n/N (%)	786/1033 (76.1)	28/54 (51.9)	674/802 (84.0)	45/99 (45.5)	35/58 (60.3)	4/20 (20.0)
*Antibiotic prescription*						
Proportion of all malaria admission cases prescribed antibiotics^a^, n/N (%)	6966/13014 (53.5)	1428/2587 (55.2)	2615/4572 (57.2)	609/1695 (35.9)	1555/2563 (60.7)	759/1597 (47.5)
Proportion with positive test prescribed antibiotics^a^, n/N (%)	4041/8050 (50.2)	1025/1902 (53.9)	1122/2046 (54.8)	369/1162 (31.8)	920/1609 (57.2)	605/1331 (45.5)
Proportion with negative prescribed antibiotics^a^, n/N (%)	1463/2338 (62.6)	368/614 (59.9)	290/464 (62.5)	172/319 (53.9)	497/718 (69.2)	136/223 (61.0)

^a^Broad spectrum antibiotics including crystalline penicillin, gentamicin, ceftriaxone, amoxicillin or chloramphenicol was prescribed

### Treatment of malaria cases

**Fig. 2:**
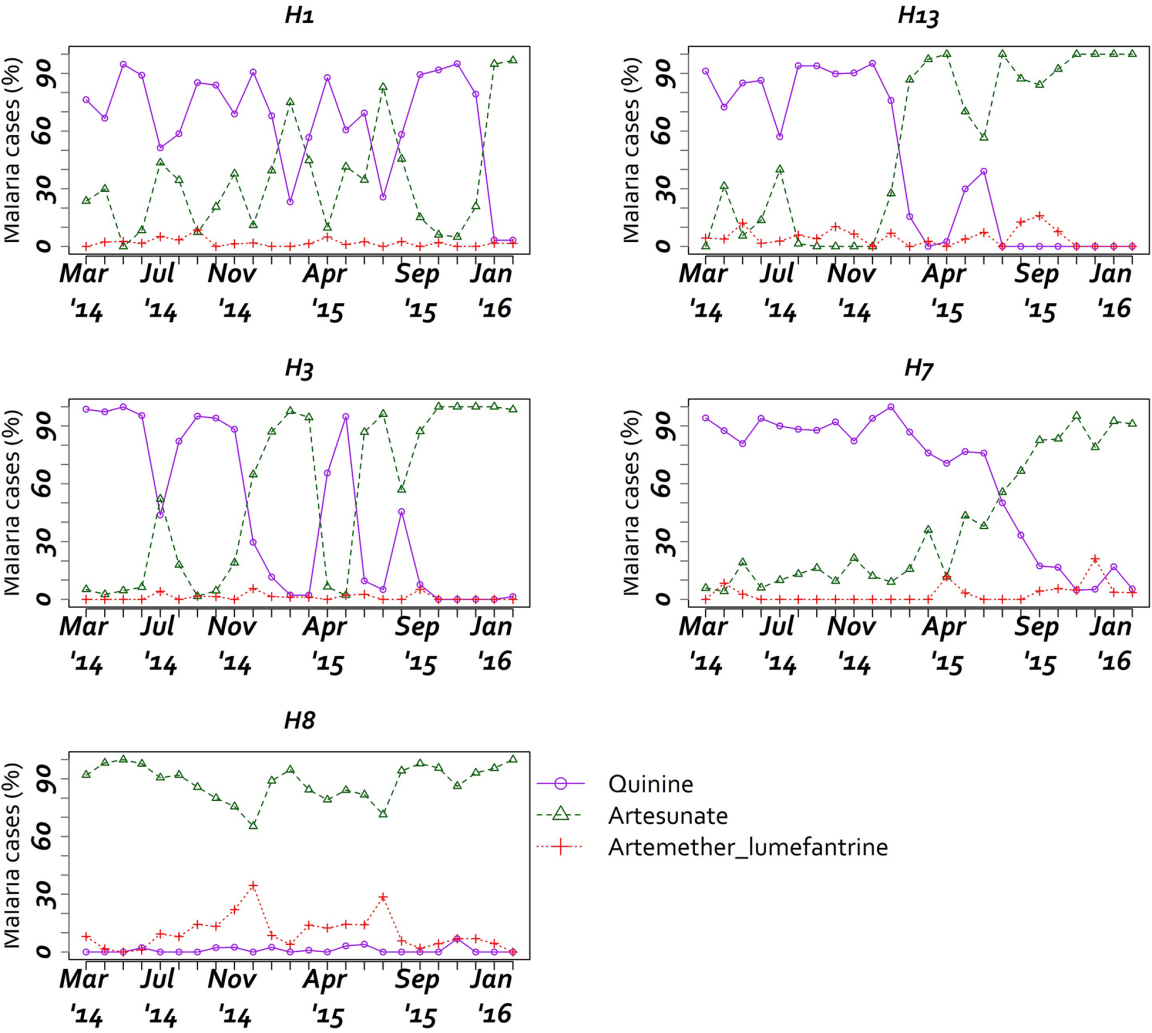
Anti-malarial drugs prescription pattern in children with admission diagnosis of malaria per hospital

A total of 6745/8050 (83.8 %) patients with a positive malaria test were clearly prescribed anti-malarials. Among the remaining 1305 (16.2 %) a total of 1120/1305 (85.8 %) had no treatment information available. Of the 2338 patients with a negative diagnostic test, 1613 (69.0 %, range across hospitals 53.0–80.2 %) were prescribed anti-malarials out of which 1065 (66.0 %) had signs or a diagnosis of severe malaria and only 37 (3.5 %) had a repeat test done with 28 (75.7 %) being positive. There were 1033 (7.9 %) children who were not tested for malaria and of these 786 (76.1 %) had anti-malarials prescribed. The anti-malarial drugs and formulations used were tablets for oral artemether-lumefantrine and injections for quinine, artemether and artesunate. Among the cases with a positive malaria test given anti-malarials on admission (n = 6745), quinine was the most prescribed drug in 3184 (47.2 %), followed by artesunate in 3108 (46.1 %) and the least prescribed drug on admission was artemether-lumefantrine in 250 (3.7 %) (Table 4). Hospitals most commonly prescribed parenteral quinine except H8 where 89.0 % were prescribed artesunate (see Fig. 2). For children with a negative malaria test given anti-malarials (n = 1613), artesunate was the most prescribed drug 845 (52.4 %) followed by quinine 597 (37.0 %).

**Table 4:** Anti-malarial drugs prescription in children with a positive malaria test across 5 Kenyan County Hospitals

Type of anti-malarial	Overall (n = 6745) (%)	Hospitals				
		H1 (n = 1642) (%)	H3 (n = 1760) (%)	H7 (n = 909) (%)	H8 (n = 1347) (%)	H13 (n = 1087) (%)
Quinine	3184 (47.2)	1075 (65.5)	946 (53.8)	587 (64.9)	13 (1.0)	563 (51.8)
Artesunate	3108 (46.1)	478 (29.1)	739 (42.0)	244 (26.8)	1199 (89.0)	448 (41.2)
Quinine and artesunate	158 (2.3)	56 (3.4)	26 (1.5)	54 (5.9)	2 (0.2)	20 (1.8)
Coartem (Artemether-lumefantrine)	250 (3.7)	33 (2.0)	18 (1.0)	19 (2.1)	124 (9.2)	56 (5.2)
Other anti-malarials^a^	45 (0.7)	0	31 (1.8)	5 (0.6)	9 (0.7)	0

^a^Either a combination of artesunate or quinine or artemether lumefantrine with artemether or artemether only prescribed

Among all malaria cases who were prescribed quinine, 59.1 % of the loading and 93.1 % of the maintenance doses were correct. Approximately two-thirds (63.8 %) of children given artesunate were prescribed the recommended dose.

Prescription patterns of anti-malarial drugs varied by hospital and by time (Fig. 2). In H1 and H3 they alternated between quinine and artesunate. Prescription of artesunate was consistently high in H8, whereas H3, H7 and H13 transitioned to use of artesunate during the monitoring period. Among the cases who were prescribed either quinine or artesunate, (n = 9840), 7598 (77.2 %) had signs suggestive of severe or complicated malaria or a diagnosis of severe malaria. Anti-malarial prescription rates for children with a negative test ranged between 15 and 100 % across the hospitals throughout the period. Antibiotics prescription among the patients testing negative and positive for malaria was 62.6 and 50.2 %, respectively (Table 3).

## Discussion

The aim of this study was to determine the investigation and treatment practices for children aged 2–59 months admitted with malaria in Western Kenya County hospitals 3 and 4 years after national and international malaria guidelines changed respectively. Documentation of clinical features of malaria (whether present or absent) was good, including the signs that define severe malaria; level of consciousness and respiratory distress [10]. This is an improvement compared to an earlier studies done in Kenya [20], Uganda [21] and in Benin [22], and is likely due to routine use of a structured Paediatric Admission Record (PAR) that guides junior clinicians to conduct a complete physical examination of sick children at admission [23, 24] associated with feedback on documentation quality given to hospitals in the form of two monthly reports [17]. Malaria laboratory testing rates when commencing inpatient treatment for clinical malaria were 92 % with recording of diagnostic test results in medical notes found in over three quarters. Findings on rates of testing and reporting are similar to some recent studies in Uganda [25] and Benin [22], but better than other studies from the region and earlier studies from Kenya [20, 23, 26–28]. Findings therefore suggest some success in adoption of the test and treat approach.

Over 80 % of malaria positive cases were clearly prescribed anti-malarial drugs. However, almost half were prescribed intravenous quinine despite the change in recommendations to use of artesunate in 2012. The trend was consistent across all the hospitals in the beginning of the period except H8 where most patients were given artesunate throughout the study period. In addition, there was a shift towards intravenous artesunate towards the end of the period in H3, H7 and H13. Similar results were reported in Uganda [25] and although the use of artesunate in 2014/2016 is unsatisfactory it is an improvement on a 2012 Kenyan survey when no inpatient malaria patients were given artesunate [23]. Informal enquiries through hospital staff indicated that the consistent prescription of artesunate in H8 was because of local efforts to ensure consistent drug availability (including local purchase). The lower rates of prescribing artesunate in other facilities were in part attributed to stock outs as well as continued difficulties changing prescribing habits.

While there is a transition from quinine to artesunate almost half of the clinical malaria cases prescribed parenteral anti-malarials had no signs of severe malaria documented (and thus were able to eat and drink). Despite this there were very low rates of giving the recommended ACT, AL to these ‘non-severe’ cases, signifying potential overuse of injectable anti-malarials in children [21]. This may be attributed to clinicians’ preference for parenteral drugs and the belief that they are ‘stronger’ [29]. On a positive note most of the dosing per body weight of both quinine and artesunate was correct, similar to a recent study in Uganda [21], but representing a considerable improvement from earlier Kenyan studies and potentially attributable to efforts to promote national paediatric guidelines in Kenya [26].

These data show high rates of presumptive treatment among children admitted with a negative malaria test or without a test done. Some reasons for this might include; associating malaria with high mortality [30], mistrust of negative test results, and concerns about missing possible malaria cases with potentially fatal consequences [30, 31]. Similar results have been reported in various studies across Africa [20, 21, 28, 30, 32–34]. The trend seems to be different from outpatient studies done immediately after the 2010 new treatment guidelines in Uganda [35] and Tanzania [36], and recent findings, that reported a decrease in prescribing anti-malarials to malaria test negative patients in Kenya [13]. Worth noting is the low rate of repeat testing of negative cases that were started on anti-malarials despite the national guidelines’ recommendation of repeat testing to confirm absence of malaria and stopping treatment if the results are still negative [10, 37]. This ‘over-treatment’ of malaria might lead to missing potentially treatable alternative causes of febrile illness with a negative malaria test that has been associated with high mortality [38].

As clinical features of malaria often overlap with other causes of febrile illness in children [39] or there might be co-infection with other diseases [40], it is important that health systems can support and that clinicians perform other laboratory investigations to identify and treat other causes of disease. Of some concern is the low use or availability of blood glucose testing in this population given that hypoglycaemia is common. The findings also revealed infrequent use in most hospitals of other tests (x-rays, microbiology tests, HIV test) to identify other causes of severe febrile illness in children admitted with clinical malaria. Anecdotally inability to conduct blood culture or lumbar puncture is a common problem underlying limited use of these investigations.

The study had some limitations. Firstly, the inclusion of patients in the analysis depended on the clinicians’ diagnosis of malaria, and further analysis relied on their documentation of testing and laboratory records. Similarly, the study relied on documentation in the case record of symptoms, signs and treatments to examine practices. The potential challenges of using such routine information that often has missing data were minimised through use of standardized record forms to record clinical information, timing data collection during discharge, data quality checks implemented within CIN, feedback to and development of partnerships with hospitals in the CIN all of which improved data availability and quality [17]. The study only captures data from five hospitals, hence generalizing results to other facilities in Kenya should be done with caution. Lastly, data on treatment preceding hospitalization were not available. Conceivably these information may have influenced some clinical decisions and practices which we assessed. The potential threat to reported findings posed by preadmission investigations not recorded in the CIN data is minimal because from our experience clinicians rarely use investigations conducted outside their facility as a basis for treatment for acute medical illnesses because these investigations are often perceived as unreliable, and also the essential investigations are readily available within facilities at minimal costs or for diseases like malaria these investigations are provided at no cost to patients. Despite these limitations, the findings from this study can provide an insight into the quality of routine inpatient malaria case management and the extent to which national and international policies are influencing practice.

## Conclusions

There is evidence of improvements in malaria case management over the initial 24 months of Clinical Information Network activities but presumptive malaria treatment for test negative results, and overuse of injectable instead of oral anti-malarials are still common practices. Three years after national policy change of the severe malaria first-line treatment to injectable artesunate, there is a gradual transition to use of artesunate. The findings suggest continued need for improvement in dissemination and implementation of guidelines for inpatient care and improved, reliable access to recommended investigations and drugs. Finally, efforts to improve clinical information could help track progress.
